# Two new species of *Dilkea* subgenus Dilkea (Passifloraceae) from Loreto, Peru

**DOI:** 10.3897/PhytoKeys.2.722

**Published:** 2011-02-11

**Authors:** Christian Feuillet

**Affiliations:** Department of Botany, MRC–166, Smithsonian Institution, P.O. Box 37012, Washington, D.C. 20013–7012, USA

**Keywords:** *Dilkea hebes*, *Dilkea nitens*, Passifloraceae, Loreto, Peru

## Abstract

Two new species of Dilkea subgenus Dilkea (Passifloraceae) are described from Loreto, Peru. Dilkea hebes Feuillet, **sp. nov.**, has leaves with elliptic to oblanceolate blades that are dull adaxially, and spherical fruits with thick walls; Dilkea nitens Feuillet, **sp. nov.**, has leaves with narrow-ovate blades that are shiny adaxially, and fruits with an apical cone and thin walls. A key to the species of subgenus Dilkea is provided.

## Introduction

Dilkea Mast. belongs in the Passifloraceae Juss. ex Roussel subfamily Passifloroideae Burnett tribe Passifloreae DC. and currently includes 12 Neotropical species including the two described below. Among the New World genera of Passifloraceae, Dilkea is a distant second to Passiflora L. with its ca. 550 species.

Dilkea is characterized by tetramerous flowers with 8 stamens, a set of 4 carpels with 4 styles fused in the basal third, an androgynophore lacking or rarely sub-null, the operculum lacking, and seeds of a peanut shape and size, while Passiflora has pentamerous flowers with 5 stamens, an androecium with 3 carpels with 3 free styles, a well–developed androgynophore, an operculum of various shapes, and seeds flattened and usually much smaller. The two other Neotropical genera each with 2–3 species are tetramerous like Dilkea, but Mitostemma Mast. has free styles and the species of Ancistrothyrsus Harms are hairy throughout and their fruits are capsular, while Dilkea species are glabrous and their fruits are baccate.

In the past few years, I came across many collections of Dilkea, a genus previously poorly collected. Six species are presently recognized in subgenus Epkia Feuillet, (2009) and are often confused with Clavija Ruiz & Pav. in the Primulaceae (APG III 2009). New species from the Guiana Shield have been described recently (Feuillet 2010a). The six species of Dilkea subg. Dilkea have similar white flowers and the useful morphological characters are mainly the position and structure of the inflorescence and the shape of the leaves and the fruits.

## Taxonomic treatment

### Dilkea subgenus Dilkea

Dilkea subgenus Dilkea is composed of lianas or climbing shrubs, or small shrubs in the case of Dilkea margaritae Cervi, with a continuous growth and thick tendrils trifid at the apex rather than shrubs or small trees with a strongly rhythmic growth and usually without tendrils in subgenus Epkia. The six species of subgenus Dilkea have been collected from Panama to Amapá (Brazil) and from Amazonian Bolivia to the Guianas.

#### Dilkea hebes

Feuillet sp. nov.

urn:lsid:ipni.org:names:77109525-1

Fig. 1 

##### Latin

Dilkea hebes foliis super hebeti, exocarpio 4–5 mm crasso, multistrato, suberis simulanti, a subgeneris Dilkea speciebus mihi notis distincta. Liana in sylva riparia inundata, in Loreto (Peruvia) crescit.

##### Type:

PERU. **Loreto:** Maynas, District of Iquitos, Río Nanay, above Bellavista, between Pampa Chica and Santa Clara, shoreline forest frequently inundated, 1 June 1976, fr., *M. Rimachi Y. 2336* (holotype : US-3393830!; isotype: IBE).

Woody climber, growth continuous, internodes subequal or gradually unequal, twigs drying brown to black; glabrous throughout. Stipules not seen. Leaf: petiole terete, reduced to the pulvinus, 3–6 mm long, swollen but not wrinkled when dry, drying nearly black; blades coriaceous, narrowly elliptic to oblanceolate, widest ca. 2/3 from base, 7.5–19 × 3.5–6.5 cm, base usually slightly acute, angle ca. 45° each side of the midrib, apex acute or round and short–acuminate, margin undulated on herbarium specimens, probably due to dorsiventrally curved midrib, adaxially dull, drying dark olive–green, abaxially dull, drying pale olive–green, midrib adaxially in a groove, abaxially strongly raised, main veins obscure, 15–20 on each side of the midrib of well–developed leaves. Inflorescences axillary; peduncle axillary, thicker than the stem, bearing 1–2 flowers; bracts thick scale-shaped, 0.5–2 mm long, long and narrow to short and wide under the same fruit; pedicel and peduncle forming a narrow cone 0.5–1.5 cm long, 0.4–0.6 cm diam. at apex under the fruit. Flowers not seen. Fruits subterminal by withering of the apical part of the stem, spherical, observed still green but well-developed, fruit wall hard, 4–5 mm thick, corky, with 1–2 seeds, partly empty with at least partially dividing membranes; seeds peanut-shaped, slightly asymmetric, 1.7 × 0.7–0.8 cm.

**Figure 1.**
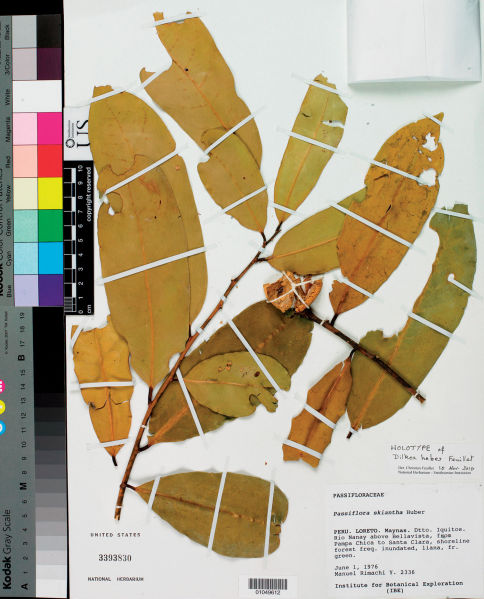
Holotype of Dilkea hebes Feuillet: *M. Rimachi Y. 2336* (US).

##### Distribution.

Endemic to the district of Iquitos, Maynas, Loreto, Eastern Peru.

The specimen labels did not give geographical coordinates for the collection localities of Dilkea hebes in Peru, Loreto, Maynas, Distr. Iquitos. The localities cited for the two specimens, “quebrada de Morropón” or “Pampa Chica”, are not in the gazetteer published by the United States Board on Geographic Names (USBGN 1989), and Santa Clara and Bella Vista are common village names in Peru. A search in the National Geospatial–Intelligence Agency database (NGA web site) on Santa Clara brought 11 occurrences in Loreto and Bella Vista / Bellavista 16. Coordinates for those are too uncertain to be presented in the specimen citations, but it is possible that they are Puerto Bellavista Nanay, port of Iquitos on the Río Nanay, ca. 3°42'S, 73°15'W, and Santa Clara de Nanay, near the far end of Iquitos airport on the bank of the Río Nanay, ca. 3°47'S, 73°20'W.

##### Ecology.

Dilkea hebes is growing in inundated riparian forest, known only from the vicinity of the type locality, ca. 90 m elev.

##### Phenology.

Fruits immature when collected in February and June.

##### Preliminary conservation status.

Dilkea hebes is known from two collections from the same district in Maynas, Loreto, Peru. The data are insufficient to assess an informed status for this species, therefore I suggest that it be classified as DD (Data Deficient) according to IUCN (2001, 2003) categories.

##### Discussion.

Dilkea hebes has a few fruit characteristics that distinguish it from all other species of Dilkea and that may be related to its frequently inundated habitat. The thick corky fruit wall, the small number of seeds leaving empty space in the fruit, and possibly the thin membranes inside the fruit increase the floatability of the fruit suggesting that the fruits may be disseminated during floods. Cork has been found in some species of Passiflora, for example on the bark of mature stems of Passiflora suberosa L. and Passiflora sexocellata Schlecht. in subg. Decaloba (DC.) Rchb. supersect. Cieca (Medic.) J.M. MacDougal & Feuillet or even on the young stems and the petioles of Passiflora phellos Feuillet in subg. Passiflora supersect. Laurifolia (Cervi) Feuillet & J.M. MacDougal. The vegetative characters are rather similar to Dilkea johannesii Barb.Rodr., which differs by its thin-walled fruit tapering at base and having an apical hard cone (cf. original illustration, reproduced from the Smithsonian Library, Feuillet 2010b).

##### Etymology.

The Latin epithet *hebes* (= dull), refers to the upper surface of the leaves that is not shiny, in contrast with Dilkea nitens Feuillet, another species from Loreto described below.

##### Paratypes.

PERU. **Loreto:** Maynas, District of Iquitos, Río Nanay, quebrada de Morropón, inundated bank, ca. 90 m, 14 Feb 1985, fr., *M. Rimachi Y. 7755* (US!; IBE).

#### Dilkea nitens

Feuillet sp. nov.

urn:lsid:ipni.org:names:77109526-1

Fig. 2 

##### Latin

Dilkea nitens *foliis anguste ovatis a generis* Dilkea *speciebus mihi notis distincta. Liana vel frutex; in Loreto (Peruvia) crescit*.

##### Type:

PERU. **Loreto:** Maynas, Pena Negra, 25 km SW of Iquitos, 3°38.6666'S, 73°20.15'W, 1 Aug 1972, fr., *T.B. Croat 18649* (holotype: US-2788507!; isotype: MO!)

“Shrub or vine” (fide *Croat 18649*), growth continuous, internodes subequal or varying gradually in size, twigs with internodes 0.5–1.5 cm long, drying pale brown; glabrous throughout. Stipules not seen. Leaf: petiole terete, 1.5–3 cm long, drying pale brown except for the basal 1/3–1/2 pulvinus, slightly swollen and drying black; blades coriaceous, narrowly ovate, 8.5–19 × 5–6 cm, widest near base, base round, apex acute or round and short–acuminate, margin undulated (in herb., probably because of the dorsiventrally curved midrib), drying pale brown, somewhat shiny, midrib dorsiventrally curved, adaxially raised, abaxially strongly raised, main veins clearly visible abaxially, 12–18 each side of the midrib of well-developed leaves. Inflorescences subterminal by abscission of the apical stem meristem; peduncle as thick as or thicker than the stem bearing it, much shorter than the petioles, ca. 5 mm long, forming apically a nearly flat surface bearing 1–6 flowers; bracts scale–like, triangular, less than 1 mm long; pedicels sub-null to 0.2 cm long. Flowers not seen. Fruits sub-spherical, 4 × 3 cm including a 3–4 mm long narrow apical cone, wall less than 1 mm thick, rigid and brittle, seen immature green; seeds not seen, brown (fide *Croat 18649*), few.

**Figure 2.**
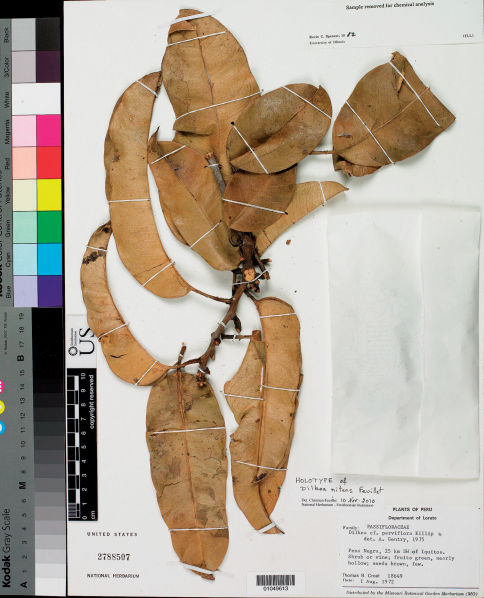
Holotype of Dilkea nitens Feuillet: *T.B. Croat 18649* (US).

##### Distribution.

Dilkea nitens is known only by the type collection from Loreto (Peru). The geographical coordinates are taken from a gazetteer (USBGN 1989).

##### Ecology.

The label does not give any information about the habitat.

##### Phenology.

It had immature fruits in August.

##### Preliminary conservation status.

Dilkea nitens is only known from the type collection from Maynas, Loreto, Peru. The data are insufficient to assess an informed status for this species, therefore I suggest that it be classified as DD (Data Deficient) according to IUCN (2001, 2003) categories.

##### Discussion.

In subgenus Dilkea where most species have oblanceolate to obovate leaf blades, Dilkea nitens is clearly identified by its narrowly ovate leaf blades, widest a quarter of the length from the base or less, a character shared only with Dilkea margaritae Cervi from Mato Grosso (Brazil) which, in contrast with Dilkea nitens, is a low shrub with 3.5–7 cm long axillary pedicels.

##### Etymology.

The epithet coming from the Latin adjective *nitens* (= shiny), refers to the upper surface of the leaves, in contrast with Dilkea hebes, another species from Loreto described above.

### Key to the species of Dilkea subg. Dilkea

**Table:** 

1.	Leaf blades narrowly ovate, widest in the basal 1/4	2
–	Leaf blades elliptic to obovate, widest in the apical 1/2	3
2.	Flowers solitary, axillary; pedicels 3.5–7 cm long	Dilkea margaritae Cervi
–	Inflorescences subterminal; peduncle capitate with few flowers, ca. 0.5 cm long; pedicels up to 0.2 cm long	Dilkea nitens Feuillet, sp. nov.
3.	Fruit wall 2–4 mm thick, corky	Dilkea hebes Feuillet, sp. nov.
–	Fruit wall about 1 mm thick, brittle	4
4.	Leaf blades membranous or chartaceous	Dilkea granvillei Feuillet
–	Leaf blades coriaceous	5
5.	Leaf blades elliptic, 5–11 × 4–7.5 cm	Dilkea clarkei Feuillet
–	Leaf blades oblanceolate to obovate, 15–28 × (5–)7–14 cm	Dilkea retusa Mast

## Corrigendum

In Feuillet (2009), the collection *J. Brandbyge & E. Asanza 31937* (AAU) was cited by mistake as a paratype under both Dilkea cuneata Feuillet and Dilkea tillettii Feuillet. The correct identification for this collection is Dilkea tillettii, as could be easily checked by looking at the scan of the specimen on the AAU web site.

## Supplementary Material

XML Treatment for Dilkea hebes

XML Treatment for Dilkea nitens
